# β Subunits Control the Effects of Human Kv4.3 Potassium Channel Phosphorylation

**DOI:** 10.3389/fphys.2017.00646

**Published:** 2017-09-01

**Authors:** Geoffrey W. Abbott

**Affiliations:** Bioelectricity Laboratory, Department of Physiology and Biophysics, School of Medicine, University of California, Irvine Irvine, CA, United States

**Keywords:** Brugada syndrome, cardiac arrhythmia, heart failure, potassium channel, ventricle

## Abstract

The transient outward K^+^ current, *I*_to_, activates early in the cardiac myocyte action potential, to begin repolarization. Human *I*_to_ is generated primarily by two Kv4.3 potassium channel α subunit splice variants (Kv4.3L and Kv4.3S) that diverge only by a C-terminal, membrane-proximal, 19-residue stretch unique to Kv4.3L. Protein kinase C (PKC) phosphorylation of threonine 504 within the Kv4.3L-specific 19-residues mediates α-adrenergic inhibition of *I*_to_ in human heart. Kv4.3 is regulated in human heart by various β subunits, including cytosolic KChIP2b and transmembrane KCNEs, yet their impact on the functional effects of human Kv4.3 phosphorylation has not been reported. Here, this gap in knowledge was addressed using human Kv4.3 splice variants, T504 mutants, and human β subunits. Subunits were co-expressed in *Xenopus laevis* oocytes and analyzed by two-electrode voltage-clamp, using phorbol 12-myristate 13-acetate (PMA) to stimulate PKC. Unexpectedly, KChIP2b removed the inhibitory effect of PKC on Kv4.3L (but not Kv4.3L threonine phosphorylation by PKC *per-se*), while co-expression with KCNE2, but not KCNE4, restored PKC-dependent inhibition of Kv4.3L-KChIP2b to quantitatively resemble previously reported effects of α-adrenergic modulation of human ventricular *I*_to_. In addition, PKC accelerated recovery from inactivation of Kv4.3L-KChIP2b channels and, interestingly, of both Kv4.3L and Kv4.3S alone. Thus, β subunits regulate the response of human Kv4.3 to PKC phosphorylation and provide a potential mechanism for modifying the response of *I*_to_ to α-adrenergic regulation *in vivo*.

## Introduction

**Figure 1:**
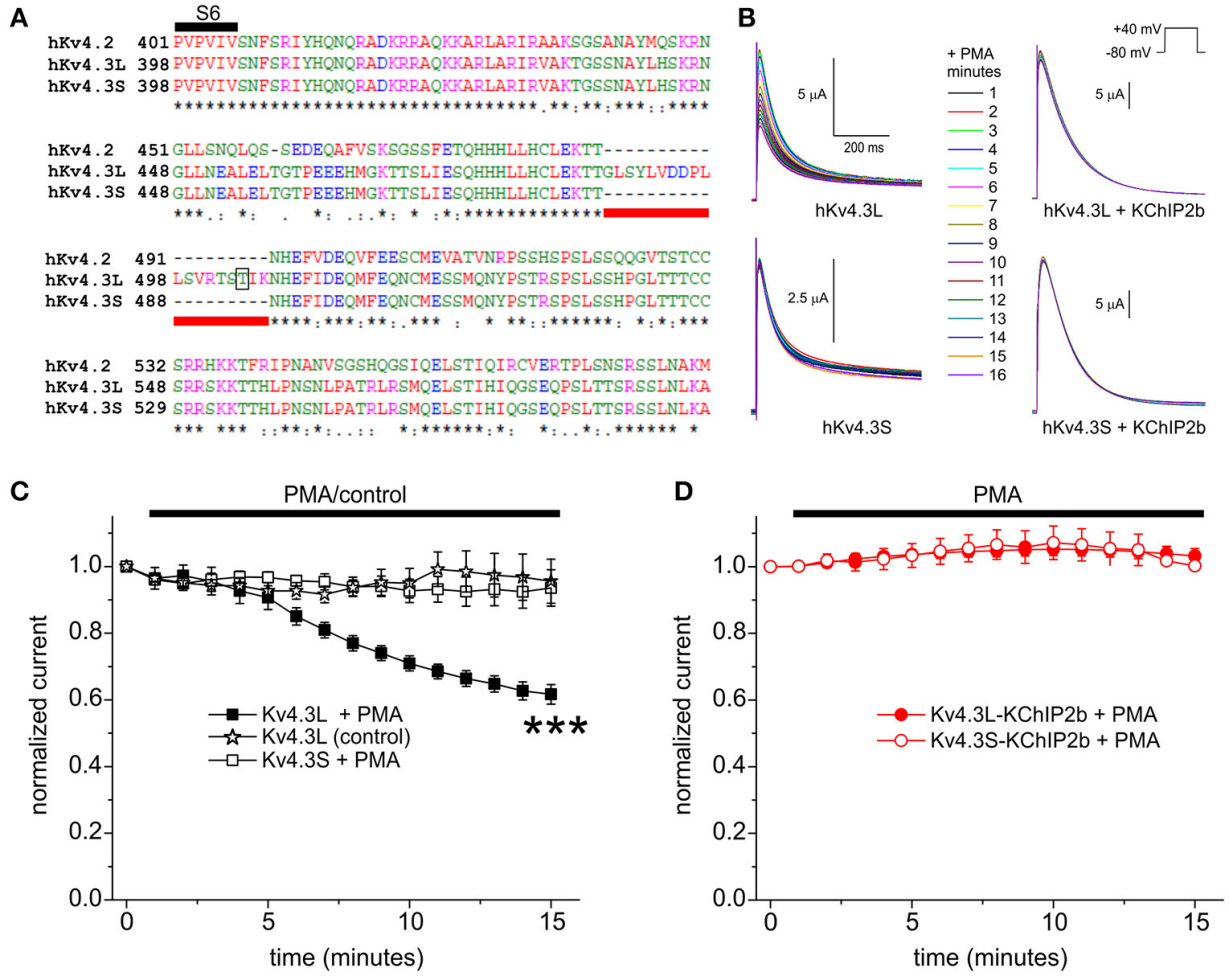
KChIP2b protects Kv4.3L from inhibition by PKC phosphorylation. **(A)** Sequence alignment (Clustal TCoffee) of the C-terminal end of human Kv4.2, Kv4.3L and Kv4.3S protein sequences, including the segment missing in Kv4.3S (underlined red) and the T504 PKC phosphorylation site (open box). ^*^Conserved in all three sequences. Adapted from Abbott (2017). **(B)** Exemplar current traces recorded during +40 mV pulses (1 per minute) in the presence of 50 nM PMA, from *Xenopus* oocytes 36–56 h after injection of 1.5 ng cRNA encoding Kv4.3L or Kv4.3S, with (*n* = 7–12) or without (*n* = 6–7) 5 ng KChIP2b. Insets: center, color-coded key to traces; upper right, voltage clamp protocol. **(C)** Mean ± SEM normalized peak current magnitude at +40 mV during incubation in 50 nM PMA or normal bath solution (control) for Kv4.3 currents recorded in the absence of KChIP2b as in **(B)** (*n* = 6–7). ^***^P < 0.001 vs. other groups at 15 min. **(D)** Mean ± SEM normalized peak current magnitude at +40 mV during incubation in 50 nM PMA (unless indicated) for Kv4.3-KChIP2b currents recorded as in **(B)** (*n* = 7–12).

In human heart, the Kv4.3 α subunit forms voltage-gated potassium (Kv) channels that generate a subthreshold-activating, rapidly inactivating Kv current (the transient outward current, or *I*_to_) important for early phase 1 repolarization of cardiac myocytes (Dixon et al., 1996; Johns et al., 1997). Kv4.3 and related α subunits Kv4.1 and Kv4.2 share relatively high sequence identity, except for a divergent additional exon only in Kv4.3 that encodes 19 amino acids within the S6-proximal cytosolic C-terminus (residues 488–506). This extra exon is commonly spliced, resulting in expression of two Kv4.3 splice variants in human tissues. The shorter form of Kv4.3, lacking this 19-residue stretch, is termed Kv4.3S; the longer form is named Kv4.3L and is otherwise identical to Kv4.3S (Figure 1A; Ohya et al., 1997; Kong et al., 1998).

Kv4.3L and Kv4.3S are each expressed in human and rat heart (Ohya et al., 1997; Kong et al., 1998). Homomeric Kv4.3L and Kv4.3S (i.e., channels formed from either α subunit alone, in the absence of β subunits) are functionally indistinguishable at baseline with respect to macroscopic current density, gating kinetics and voltage dependence. However, the two isoforms are functionally distinct in at least two respects. First, Kv4.3L contains a consensus threonine phosphorylation site encoded by its additional exon, not present in Kv4.3S. Protein kinase C (PKC) phosphorylation of T504 within this consensus site mediates inhibition of human Kv4.3L, facilitating physiologically important α-adrenergic regulation of cardiac *I*_to_ (Po et al., 2001). In contrast, others found that for rat Kv4.3, PKC stimulation (10 nM PMA for 30 min) inhibited both long and short forms, T504A-Kv4.3L-indepedently, with the only isoform-dependent difference being differentially altered closed-state inactivation (decreasing it in Kv4.3S, increasing it in Kv4.3L; Xie et al., 2009).

Second, co-expression with β subunits can uncover functional differences in Kv4.3L vs. Kv4.3S. Potassium channel interacting protein 2 b (KChIP2b) is a member of the KChIP family of β subunits, several of which co-assemble with Kv4 (An et al., 2000) and other Kv α subunits (Li et al., 2005). KChIP2b is highly expressed in human heart, where it forms channels with Kv4.3. In studies of human, rat, and/or ferret subunits, KChIP2b augmented Kv4.3 current, altered its pharmacology (Bett et al., 2006), slowed its inactivation, accelerated its recovery from inactivation, positive-shifted its voltage dependence of steady-state inactivation (Patel et al., 2002, 2004; Wang et al., 2002), and promoted non-inactivated closed states in 2 mM extracellular K^+^ (Amadi et al., 2007). Strikingly, examining human subunits, KChIP2b augmented Kv43L two-fold, but Kv4.3S four-fold, under comparable expression conditions in *Xenopus* oocytes studies; and unlike homomeric channels, Kv4.3L-KChIP2b has ~40% slower inactivation than Kv4.3S-KChIP2b (Abbott, 2017). Kv4 channels are also regulated by KCNE subunits—ubiquitously expressed, promiscuous, single transmembrane-spanning β subunits (Abbott, 2015, 2016b,c). The arrhythmogenic effects of their gene deletion in mice and/or inherited human mutations support the necessity for KCNE and KChIP regulation of Kv4 channels in mammalian heart (Kuo et al., 2001; Deschenes and Tomaselli, 2002; Radicke et al., 2006). For example, Brugada syndrome, a potentially lethal cardiac rhythm disturbance, can be caused by increased ventricular *I*_to_ resulting from gain-of-function mutations not only in *KCND3* (which encodes Kv4.3; Giudicessi et al., 2011), but also in *KCNE3* (Delpon et al., 2008) and *KCNE5* (Ohno et al., 2011).

α-Adrenergic inhibition of cardiac myocyte *I*_to_, which occurs via PKC phosphorylation of Kv4.3L-T504 in the case of human Kv4.3, has been recorded in isolated rat, rabbit, and human atrial and/or ventricular cardiac myocytes and may contribute to regulation of action potential duration *in vivo* (Apkon and Nerbonne, 1988; Braun et al., 1990; Fedida et al., 1990; Po et al., 2001). It is thought that Kv4.3 is regulated in human heart by KChIP2 (and in particular the preeminent isoform in human heart, KChIP2b) and one or more of the KCNEs (Kuo et al., 2001; Deschenes and Tomaselli, 2002; Radicke et al., 2006). Indeed, individual cardiac Kv4 channel complexes may each incorporate both KCNE proteins and KChIPs in cardiac myocytes (Radicke et al., 2006; Liu et al., 2008; Levy et al., 2010). This, together with the functional divergence of Kv4.3L and Kv4.3S under certain conditions, potentially endows *I*_to_ with temporally and spatially diverse functional properties dependent upon relative subunit and splice isoform expression levels. The ability of PKC phosphorylation, and β subunit co-assembly, to independently differentiate physiologically important aspects of Kv4.3L and Kv4.3S function raises the question of whether β subunits can alter the functional responses of Kv4.3 to PKC phosphorylation. Here, this question was addressed utilizing wild-type and mutant human channel subunits co-expressed in the *Xenopus laevis* oocyte expression system.

## Materials and methods

### *Xenopus laevis* oocyte channel subunit cRNA preparation and injection

cRNA transcripts encoding hKv4.3L, hKv4.3S and hKChIP2b were generated by *in vitro* transcription (T7 polymerase mMessage mMachine kit, Thermo Fisher Scientific), after vector linearization, from cDNA sub-cloned into plasmids (a kind gift of Dr. Steve A. N. Goldstein, Brandeis University, Waltham, MA) incorporating *X. laevis* β-globin 5′ and 3′ UTRs flanking the coding region to enhance translation and cRNA stability. T504A and T504D Kv4.3L mutants were made using a QuikChange Site-Directed Mutagenesis kit (Agilent, Santa Clara, CA) per the manufacturer's protocol and confirmed by sequencing. Human KCNE2 and KCNE4L were also transcribed from cDNA templates incorporating *X. laevis* β-globin 5′ and 3′ UTRs. cRNA was quantified by spectrophotometry. Defolliculated stage V and VI *X. laevis* oocytes (Ecocyte Bioscience, Austin, TX) were injected with one, two or three of the subunit cRNAs as follows: 1.5 ng of Kv4.3L or Kv4.3S; with or without 5 ng of KChIP2b, with or without 5 ng of KCNEx. Oocytes were incubated at 16°C in SBB solution (Ecocyte) containing penicillin and streptomycin, with daily washing, for 2–3 days prior to two-electrode voltage-clamp (TEVC) recording.

### TEVC

TEVC recordings were made at room temperature using an OC-725C amplifier (Warner Instruments, Hamden, CT) with pClamp8 software (Molecular Devices, Sunnyvale, CA), from *X*. laevis oocytes in a small-volume oocyte bath (Warner Instruments), visualized with a dissection microscope. The TEVC bath solution was (in mM): 96 NaCl, 4 KCl, 1 MgCl_2_, 1 CaCl_2_, 10 HEPES (pH 7.6); bath chemicals, including PMA and the biologically inactive phorbol 4α-Phorbol 12,13-didecanoate (PDD) were from Sigma-Aldrich (St. Louis, MO). TEVC pipettes were of 1–3 MΩ resistance when filled with 3 M KCl. Currents were recorded at 40 mV for fitting inactivation kinetics, and (at 1 min intervals) to quantify response to bath-applied PMA, PDD, or control solutions. For quantifying steady-state inactivation, oocytes were held at −100 mV and then prepulsed to voltages between −120 and 0 mV, each followed by a tail pulse to +40 mV. To quantify rate of recovery from inactivation, oocytes were double-pulsed to +40 mV with variable interpulse recovery periods (10–5,000 ms) at −120 mV, and the magnitude of the second current peak compared to that of the first peak for each pair. TEVC data analysis was with Clampfit (Molecular Devices) and Origin 6.1 (OriginLab Corp., Northampton, MA) software. Values are stated as mean ± SEM. Steady-state inactivation plots of fraction of available channels vs. voltage were plotted vs. prepulse voltage and fitted with a single Boltzmann function according to:

$$\begin{array}{l} g=\left({\text{A}}_{1}-{\text{A}}_{2}\right)/\left\{1+\text{exp}\left[\left({\text{V}}_{1/2}-\text{V}\right)/{\text{V}}_{\text{s}}\right]\right\}+{\text{A}}_{2}, \end{array}$$

where *g* is the normalized tail conductance, A_1_ is the initial value at −∞, A_2_ is the final value at +∞, V_1/2_ is the half-maximal voltage of activation and V_s_ the slope factor. Current decay arising from channel inactivation curves was fitted with a standard (zero-shift) single (where possible) or double exponential decay function with Chebyshev 4-point smoothing filter. Inactivation recovery kinetics were fitted from mean normalized fractional recovery currents to a two-phase exponential association equation:

$$\begin{array}{l} y=y_{0}+A_{1}\left(1-e^{-x/t_{1}}\right)+A_{2}\left(1-e^{-x/t_{2}}\right) \end{array}$$

and for cases in which iterative fitting yielded identical τ values, a single exponential fit was reported. Because mean inactivation recovery curves were fitted to improve fit, these data are reported as a value with no standard error, but rather a *R*^2^-test for goodness of fit. In all other cases, values are reported with standard error of the mean. Where informative, currents were compared with one another using student's *t*-test to assess statistical significance (*P* < 0.05). For multiple comparisons, ANOVA was performed, followed by a *post-hoc* Tukey's HSD test.

### Western blotting

Kv4.3L was expressed alone or with KChIP2b in *Xenopus* oocytes for 3 days, after which the oocytes were transferred to storage solution containing PMA (50 nM) for 30 min at 16°C. Following this, the oocytes were lysed in buffer composed of 1% IGEPAL, 0.1% SDS, 50 mM Tris (pH 8.0), 150 mM NaCl, phosphatase inhibitor (Sigma) and a protease-inhibitor cocktail tablet (Thermo Fisher, Waltham, MA), rotated end-over-end at 4°C for 1 h, centrifuged at 10,000 rpm for 15 min at 4°C, and then the supernatants were immunoprecipitated using overnight incubation at 4°C with a rabbit polyclonal Kv4.3 antibody (1/100 dilution) (Sigma) followed by 2 h incubation at 4°C with protein A/G agarose beads (Pierce, Rockford, IL). After bead washing, samples were heated in SDS loading buffer with reducing agent for 10 min at 70°C before being separated by SDS-PAGE. Proteins were transferred to PVDF membranes, blocked with 5% fish-skin gelatin, and then probed with mouse monoclonal antibodies (1/000 dilution) raised against epitopes on Kv4.3 (NeuroMabs, Davis, CA) or phosphothreonine (Sigma). Bands were visualized via HRP-conjugated goat anti-mouse secondary antibody (1/5,000) (Bio-Rad, Hercules, CA) and a chemiluminescent HRP substrate (Pierce).

## Results

### KChIP2b prevents inhibition of Kv4.3 by PKC

As previously reported (Po et al., 2001), and recapitulated here, bath application of PMA (50 nM) to cRNA-injected *X. laevis* oocytes, to stimulate PKC activity, inhibited Kv4.3L channel activity, quantified by measuring peak current at +40 mV (by 44 ± 3% after 15 min) but did not affect Kv4.3S current magnitude (Figures 1B,C). Neither did Kv4.3L currents run down in the absence of PMA (Figure 1C). Strikingly, however, co-expression of KChIP2 eliminated PKC inhibition of Kv4.3L, and left Kv4.3S unaffected (Figure 1D). It was technically possible that PMA was causing a concomitant increase in, e.g., Kv4.3L-KChIP2 surface expression, superimposing upon and canceling out a still-present Kv4.3L-T504 phosphorylation-dependent decrease in channel activity, so T504 was next mutated to alanine. If PMA caused a KChIP2b-dependent increase in Kv4.3 current that was T504-independent, and in fact T504-dependent inhibition was still occurring in the presence of KChIP2b, then the Kv4.3L-T504A-KChIP2b currents would be expected to increase upon incubation with PMA. This, however, was not the case. Thus, there was still no effect of PMA on T504A-Kv4.3L-KChIP2, suggesting KChIP2 either prevented Kv4.3L-T504 phosphorylation or its functional effects. Neither did biologically inactive phorbol, PDD, inhibit Kv4.3L or Kv4.3S current when co-expressed with KChIP2b, suggesting against the possibility that an off-target, current-potentiating effect of PMA was canceling out Kv4.3L inhibition in the presence of KChIP2b (Supplementary Figure 1).

### KChIP2b does not prevent PKC threonine phosphorylation of Kv4.3L

**Figure 2:**
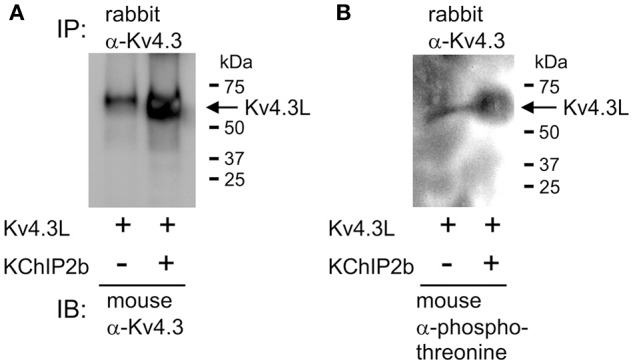
KChIP2b does not prevent Kv4.3L threonine phosphorylation. **(A)** Western blot result showing hKv4.3L protein immunoblot (IB) with mouse monoclonal anti-Kv4.3 antibody, following immunoprecipitation (IP) with rabbit polyclonal anti-Kv4.3 antibody, 3 days after Kv4.3 cRNA injection in *Xenopus* oocytes, with or without co-injected KChIP2b cRNA. Oocytes were incubated for 30 min in 50 nM PMA prior to lysis. Representative of two independent experiments. **(B)** Western blot on similar samples as in **(A)** but using mouse monoclonal anti-phosphothreonine antibody for IB. Representative of two independent experiments.

Kv4.3L was expressed alone or with KChIP2b in *Xenopus* oocytes for 3 days, PMA-treated for 30 min, and then the oocytes lysed and Kv4.3L immunoprecipitated using rabbit polyclonal anti-Kv4.3 antibody. KChIP2b increased total Kv4.3L protein expression (assessed by western blot using a mouse monoclonal anti-Kv4.3 antibody), as previously observed in CHO cell expression studies (Takimoto et al., 2002; Figure 2A). This also corresponds well to the increased Kv4.3 current observed upon KChIP2b co-expression, as shown in Figures 1B, **4B**, and previously widely reported (Bett et al., 2006). Importantly, KChIP2b co-expression equally increased the threonine phosphorylated form of Kv4.3L (Figure 2B). Therefore, despite nullifying PKC-induced Kv4.3L inhibition, KChIP2b did not prevent Kv4.3L threonine phosphorylation.

### KChIP2b alters effects of T504 mutations on Kv4.3 inactivation

**Figure 3:**
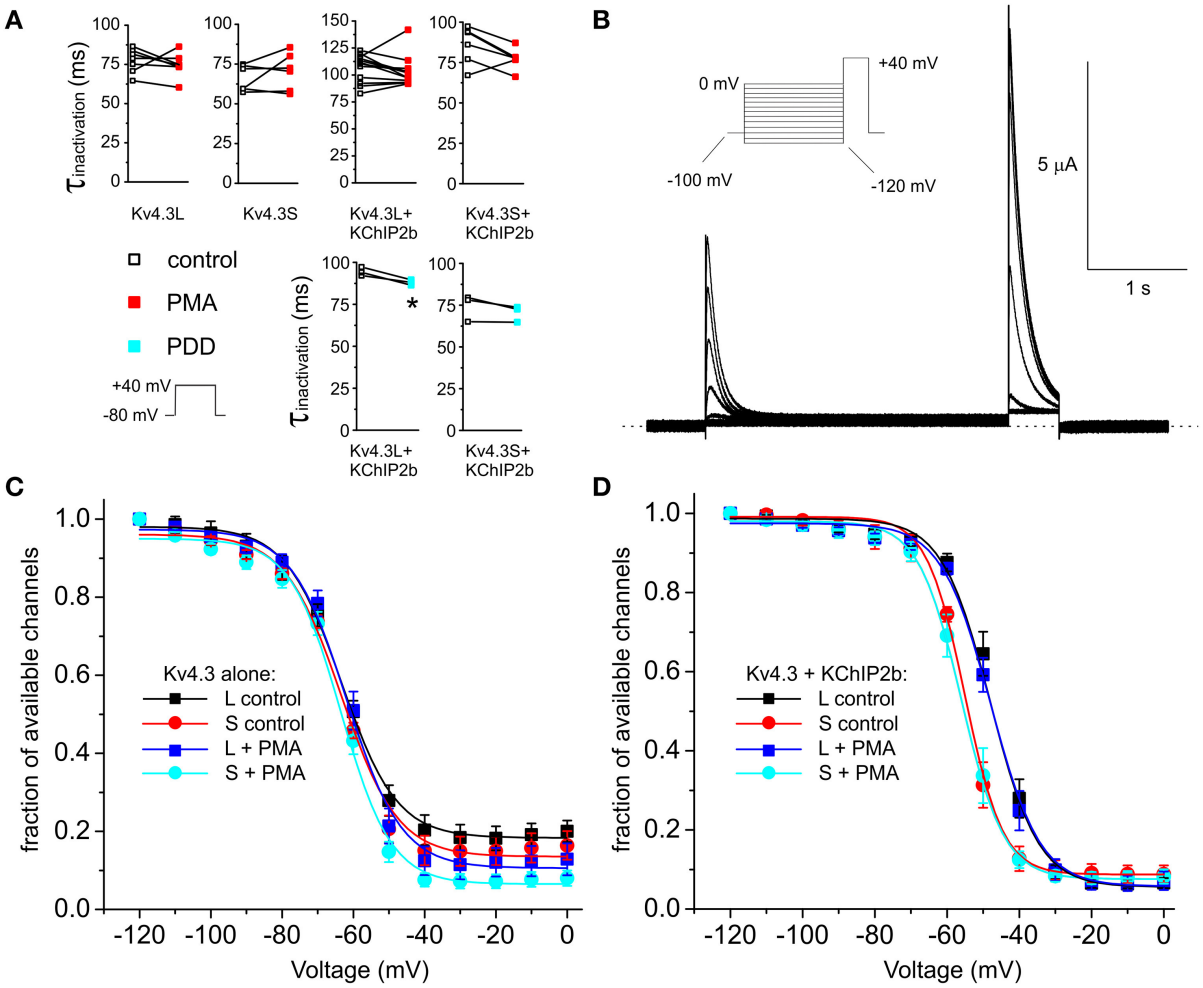
PKC stimulation has no effects on Kv4.3 and Kv4.3-KChIP2b inactivation rate and voltage dependence. **(A)** Inactivation rates at +40 mV (τ of single exponential fit) for oocytes expressing Kv4.3L and Kv4.3S alone or with KChIP2b, before and after 15 min incubation with 50 nM PMA (*n* = 6–12) or PDD (*n* = 3). Upper inset: voltage protocol. ^*^P < 0.05. **(B)** Exemplar current trace recorded from a *Xenopus* oocyte expressing Kv4.3L-KChIP2b using the steady-state inactivation protocol (upper left inset). Zero current level indicated by dashed line. **(C)** Mean ± SEM fraction of available channels/voltage relationship for Kv4.3L vs. Kv4.3S, with/without 15 min pre-incubation in 50 nM PMA; currents recorded as in **(B)**; *n* = 6–8. **(D)** Mean ± SEM fraction of available channels/voltage relationship for Kv4.3L-KChIP2b (*n* = 12–13) vs. Kv4.3S-KChIP2b (*n* = 5–9), with/without 15 min pre-incubation in 50 nM PMA; currents recorded as in **(B)**.

Incubation with 50 nM PMA for 15 min to stimulate PKC activity did not alter Kv4.3L or Kv4.3S inactivation rate. A statistically significant trend toward increased inactivation rate in the presence of KChIP2b was observed when PMA was substituted with PDD, indicating this effect was independent of PKC stimulation and may arise from repetitive pulsing or prolonged bath incubation (Figure 3A). Examining steady-state inactivation using a double-pulse protocol (Figure 3B), PMA altered neither the midpoint of voltage dependence of steady-state inactivation, nor the fraction of available homomeric Kv4.3L and Kv4.3S channels at maximal inactivation (*P* > 0.05 with vs. without PMA; Figure 3C). KChIP2b negative-shifted (by 7 mV) the voltage dependence of Kv4.3S inactivation compared to Kv4.3L-KChIP2b, independent of PKC activation (Figure 3D), as we previously reported (Abbott, 2017), but in the presence of KChIP2, PMA altered neither the voltage dependence of steady-state inactivation, nor the fraction of available channels (Figure 3D).

**Figure 4:**
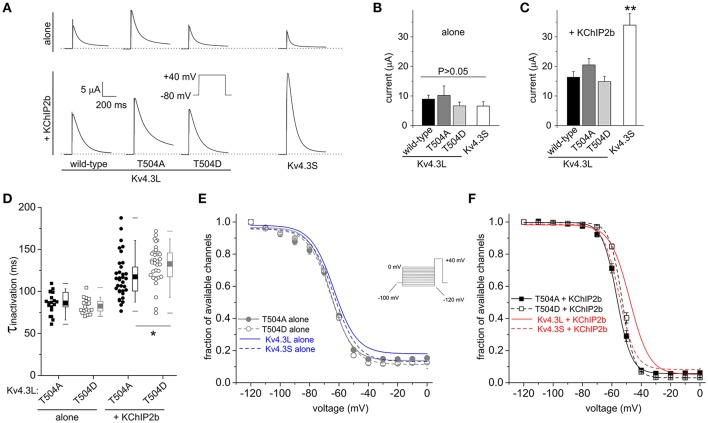
Kv4.3L T504 mutations alter inactivation rate and voltage dependence only with KChIP2b co-expression. **(A)** Exemplar current traces recorded at +40 mV from *Xenopus* oocytes expressing Kv4.3L (wild-type, T504A or T504D) or Kv4.3S, alone or with KChIP2b (voltage protocol inset). Zero current level indicated by dashed line. **(B)** Mean ± SEM peak currents at +40 mV for oocytes expressing Kv4.3 variants as in **(A)**, in the absence of KChIP2b (*n* = 8–15). **(C)** Mean ± SEM peak currents at +40 mV for oocytes expressing Kv4.3 variants as in **(A)**, with KChIP2b (*n* = 21–25). ^**^P < 0.01 vs. other +KChIP2b groups. **(D)** Box plots showing individual and mean ± SEM values for τ of inactivation at +40 mV of T504A and T504D Kv4.3L, with (*n* = 34) vs. without (*n* = 17–21) KChIP2b, recorded as in **(A)**. ^*^P < 0.05. **(E)** Mean ± SEM fraction of available channels/voltage relationship for T504A and T504D Kv4.3L vs. Kv4.3S in the absence of KChIP2b (*n* = 10–13), using the steady-state inactivation protocol (inset). Curves for wild-type Kv4.3 subunits without KChIP2b (from Figure 3) included for comparison (blue lines as indicated). **(F)** Mean ± SEM fraction of available channels/voltage relationship for T504A and T504D Kv4.3L vs. Kv4.3S, with KChIP2b (*n* = 26–31), using the steady-state inactivation protocol as in **(E)**. Curves for wild-type Kv4.3 subunits with KChIP2b (from Figure 3) included for comparison (red lines as indicated).

Neither T504A nor the constitutive phosphorylation mimic T504D had statistically significant baseline effects on Kv4.3L peak current or its two-fold upregulation by KChIP2b. However, for channels in the absence of KChIP2b, there was a (statistically not significant) ~25% lower current magnitude for Kv4.3L-T504D compared to wild-type Kv4.3L (*P* > 0.05), matching the smaller current magnitude observed for Kv4.3S (Figures 4A,B). This effect was similar to effects of T504 phosphorylation observed for wild-type Kv4.3L alone channels with PMA incubation (Figure 1). Furthermore, in the presence of KChIP2b, Kv4.3L-T504D current magnitude was only 8% less than that of wild-type (Figures 4A,C), consistent with KChIP2b greatly reducing effects of T504 phosphorylation on current magnitude, as found using PMA incubation (Figure 1). KChIP2b co-expression upregulated Kv4.3S four-fold, recapitulating this splice-dependent variation in augmentation we previously described (Abbott, 2017), and indicating that T504 is not the crucial residue in this differential (Figures 4A,C). Kv4.3L inactivation rate was slowed by 13 vs. 20% by the T504D substitution, compared to T504A Kv4.3L vs. wild-type Kv4.3L, respectively, but only when KChIP2 was co-expressed (Figure 4D; and compare to wild-type data in Figure 3A). Neither point mutation altered the voltage dependence of homomeric Kv4.3L channel steady-state inactivation (Figure 4E). In contrast, the voltage dependence of steady-state inactivation of both T504A-Kv4.3L-KChIP2b and T504D-Kv4.3L-KChIP2b channels was negative-shifted compared to that of wild-type Kv4.3L-KChIP2b channels, such that it mimicked the voltage-dependence of Kv4.3S-KChIP2b (Figure 4F).

### PKC stimulation differentially speeds Kv4.3s vs. Kv4.3L-KChIP2 inactivation recovery

**Figure 5:**
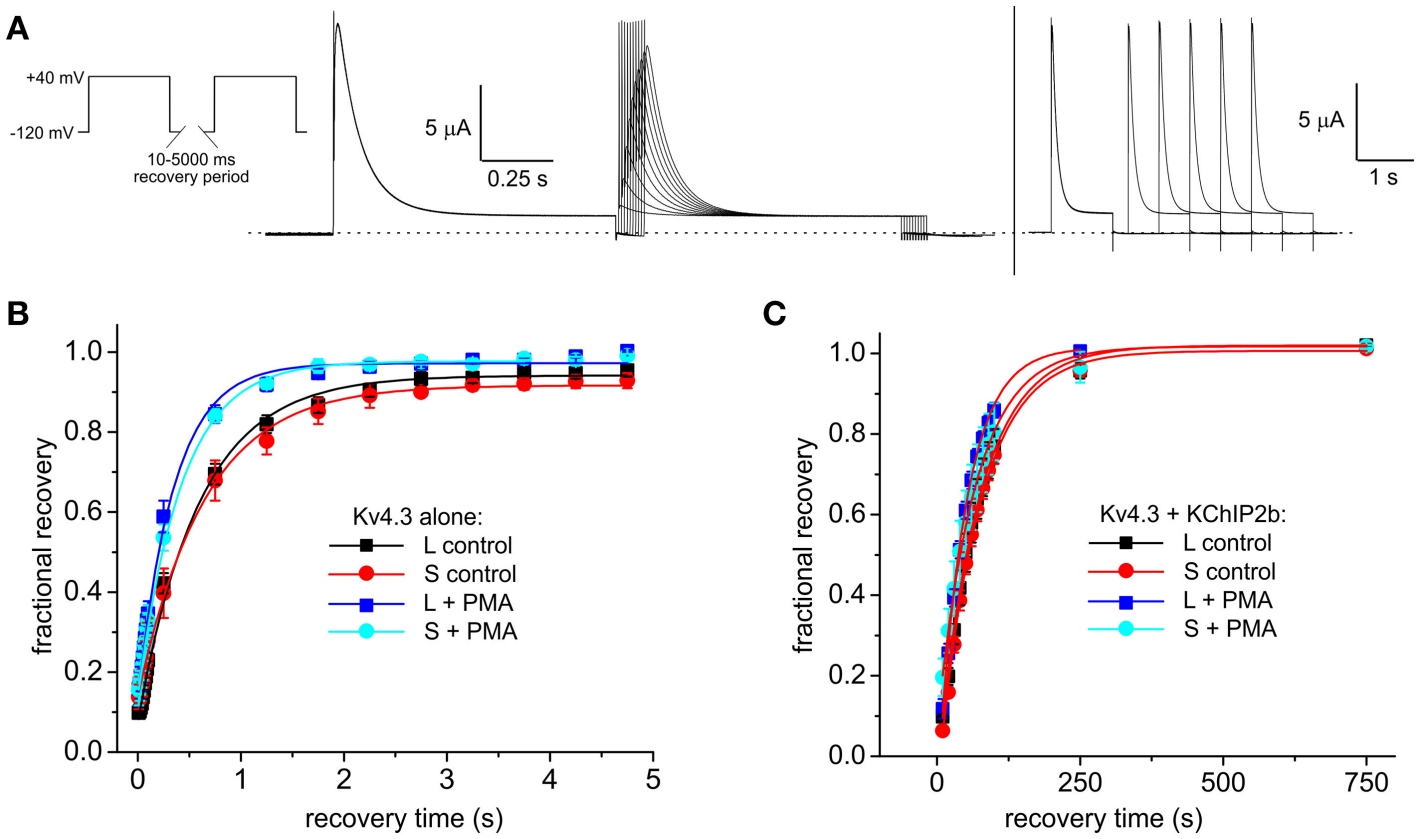
KChIP2b regulates the effects of PKC stimulation on Kv4.3 inactivation recovery. **(A)** Exemplar current traces recorded using short (left) and long (right) inactivation recovery protocols (left inset), from a *Xenopus* oocyte expressing Kv4.3L and KChIP2b. **(B)** Mean ± SEM fractional current recovery plotted from traces recorded as in **(A)** for homomeric Kv4.3L or Kv4.3S in normal bath solution (control) or after 15 min in bath solution containing 50 nM PMA; *n* = 4–5. **(C)** Mean ± SEM fractional current recovery plotted from traces recorded as in **(A)** for Kv4.3L (*n* = 11–12) or Kv4.3S (*n* = 5–10) with KChIP2b in normal bath solution (control) or after 15 min in bath solution containing 50 nM PMA.

Recovery from inactivation, as quantified by a double-pulse protocol of fixed-voltage pulses with variable interpulse interval times (Figure 5A), was similar at baseline for Kv4.3L and Kv4.3S alone (as previously reported, Abbott, 2017), but speeded (by 35–41%) for either splice variant by PMA stimulation of PKC activity (Figure 5B). When co-expressed with KChIP2, PMA speeded inactivation recovery of Kv4.3L by 23%, but Kv4.3S by a nominal 6% (Figure 5C). The non-PKC-stimulating phorbol ester, PDD, had no effect on Kv4.3L-KChIP2b recovery rate (Supplementary Figure 2). Values for curve fits for all parameters, conditions, and subunit combinations tested are provided in Table 1 (steady-state inactivation) and Table 2 (recovery from inactivation).

**Table 1:** Steady-state inactivation parameters.

**Subunits**	**V_0.5_ (mV)**	**Slope (1/mV)**	***R*^2^**
Kv4.3L (control)	−63.3 ± 0.97	7.8 ± 0.94	0.998
Kv4.3S (control)	−63.4 ± 0.71	7.9 ± 0.59	0.994
Kv4.3L-KChIP2b (control)	−48.0 ± 0.89	6.7 ± 0.49	0.998
Kv4.3S-KChIP2b (control)	−55.0 ± 0.74	5.3 ± 0.66	0.999
Kv4.3L (PMA)	−61.9 ± 1.20	7.7 ± 0.82	0.996
Kv4.3S (PMA)	−63.5 ± 0.78	7.1 ± 0.59	0.997
Kv4.3L-KChIP2b (PMA)	−47.9 ± 0.95	6.8 ± 0.55	0.996
Kv4.3S-KChIP2b (PMA)	−56.0 ± 1.06	5.9 ± 0.60	0.9995
T504A-Kv4.3L (control)	−65.5 ± 0.42	7.1 ± 0.36	0.996
T504D-Kv4.3L (control)	−65.2 ± 0.40	7.7 ± 0.31	0.997
T504A-Kv4.3L-KChIP2b (control)	−56.6 ± 0.49	4.8 ± 0.27	0.9998
T504D-Kv4.3L-KChIP2b (control)	−53.0 ± 0.34	4.2 ± 0.16	0.9999

*Fits were made to mean data and therefore P-values are not available for comparisons between groups, but R^2^-values are given to indicate goodness of fit. Graphs and n-values are given in figures and figure legends*.

**Table 2:** Inactivation recovery kinetics.

**Subunits (treatment)**	**τ_fast_ (ms)**	**A_fast_**	***R*^2^**
Kv4.3L (control)	595.6 ± 33	0.87 ± 0.01	0.999
Kv4.3S (control)	648 ± 31	0.78 ± 0.01	0.998
Kv4.3L-KChIP2b (control)	65.7 ± 1.9	1.01 ± 0.02	0.998
Kv4.3S-KChIP2b (control)	70.8 ± 2.6	1.09 ± 0.01	0.999
Kv4.3L (PMA)	350 ± 17	0.84 ± 0.01	0.998
Kv4.3S (PMA)	422 ± 23	0.84 ± 0.02	0.998
Kv4.3L-KChIP2b (PMA)	50.3 ± 1.2	1.11 ± 0.01	0.999
Kv4.3S-KChIP2b (PMA)	66.4 ± 7.9	0.95 ± 0.05	0.999
Kv4.3L-KChIP2b (PDD)	67.6 ± 2.2	1.07 ± 0.02	0.999

*Fits were made to mean data using a single exponential fit corresponding to τ_fast_ in the absence of KChIP2 and to the entire recovery period in the presence of KChIP2. P-values are not available for comparisons between groups as they were fit to mean data, but R^2^-values are given to indicate goodness of fit. Graphs and n-values are given in figures and figure legends*.

### KCNE2 restores PKC-dependent inhibition of Kv4.3L-KChIP2 activity

**Figure 6:**
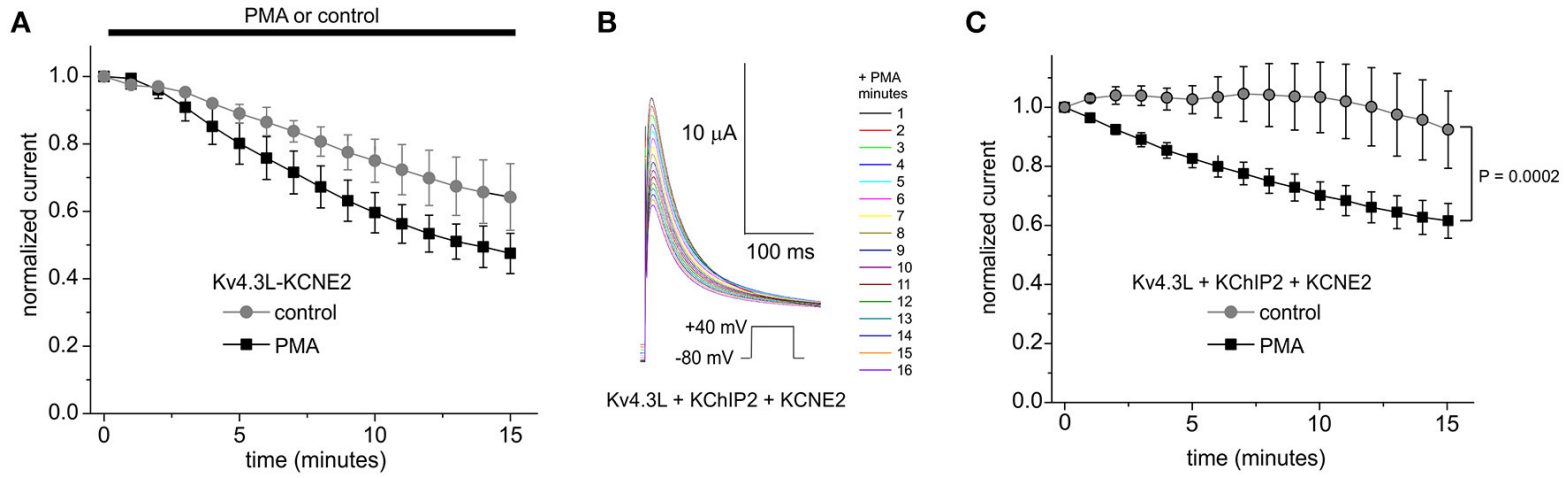
KCNE2 restores the PKC sensitivity of Kv4.3L-KChIP2b activity. **(A)** Mean ± SEM normalized peak current magnitude at +40 mV (1 pulse per minute) during incubation in 50 nM PMA (*n* = 11–12) or normal bath solution (control; *n* = 5) for Kv4.3L with KCNE2. **(B)** Exemplar current traces recorded during +40 mV pulses (1 per minute) in the presence of 50 nM PMA, from *Xenopus* oocytes expressing encoding Kv4.3L with KCNE2 and KChIP2b. Insets: center, color-coded key to traces; lower left, voltage clamp protocol. **(C)** Mean ± SEM normalized peak current magnitude at +40 mV during incubation in 50 nM PMA (*n* = 8) or normal bath solution (control; *n* = 5) for cells as in **(B)**.

Kv4.3 is thought to be regulated by KCNE subunits in cardiac myocytes. In healthy human ventricles, transcripts for all five KCNEs have been detected, the highest expression being reported for KCNE4 and then KCNE2 (Radicke et al., 2006). KCNE4L exerts inhibitory effects on either Kv4.3 splice variant (Abbott, 2016a,d, 2017). Here, the residual current for Kv4.3L-KCNE4L but not Kv4.3S-KCNE4L was decreased by PMA, although this effect did not quite reach statistical significance because the low current densities increased relative pulse-to-pulse variability (*P* = 0.11; Supplementary Figure 3A). KCNE2 introduced rundown to Kv4.3L currents, independent of PMA. PMA, nevertheless, produced an additional inhibitory effect over 15 min, only in Kv4.3L-KCNE2 channels (Figure 6A). Strikingly, KCNE2 co-expression restored the inhibitory effect of PMA on Kv4.3L-KChIP2b activity (*P* = 0.0002; Figures 6B,C). This was not PMA-independent rundown, which did not occur in channels containing KChIP2 (Figure 6C). In contrast, Kv4.3S-KCNE2 currents did not exhibit additional PMA-mediated inhibition beyond rundown (Supplementary Figure 3B). Furthermore, Kv4.3S-KChIP2b-KCNE2 showed neither rundown nor PMA-mediated inhibition (Supplementary Figures 3C,D).

## Discussion

The two known human Kv4.3 splice variants, Kv4.3L and Kv4.3S, were previously thought to function similarly at baseline, but differently with respect to their regulation by PKC, because of PKC phosphorylation of T504 in the C-terminal 19-residues that are found only in Kv4.3L (Po et al., 2001; Xie et al., 2009). Yet, the author recently found that co-expression with KChIP2b or KCNEs co-expression reveals important functional differences between Kv4.3L and Kv4.3S, namely that KChIP2 favorably augments Kv4.3S vs. Kv4.3L activity (four- vs. two-fold), and that Kv4.3S-KChIP2b channels are faster inactivating, and exhibit negative-shifted steady-state inactivation, compared to Kv4.3L-KChIP2b (Abbott, 2017).

### *I*_to_ regulation by PKC varies with subunit composition

The major findings of the current study are that KChIP2b removes the inhibitory effect of PKC on Kv4.3L (but not Kv4.3L threonine phosphorylation *per-se* by PKC), and that co-expression with KCNE2, but not KCNE4, restores the inhibitory effect of PKC. In addition, PKC accelerated recovery from inactivation of Kv4.3L-KChIP2b channels and, interestingly, both Kv4.3L and Kv4.3S homomeric channels. KChIP2b is the predominant KChIP2b isoform in human heart and is considered an obligate partner for Kv4.3 in mammalian heart. KCNE2 has been mooted as a possible partner among other KCNEs for Kv4.3 in human heart, as has KCNE4, which co-localizes with Kv4.3 in human atrial tissue (Abbott, 2016d). Because α-adrenergic stimulation has been shown to inhibit native human cardiac myocyte *I*_to_ via PKC phosphorylation (Po et al., 2001), it is thus likely that a significant fraction of native ventricular Kv4.3 channel complexes contain both KChIP2b and KCNE2 (which is the second-highest expressed KCNE subunit in human ventricular myocytes, after KCNE4), as expression with KChIP2b alone would prevent inhibition by PKC. This further reinforces the idea that KCNE2 is highly influential in regulating repolarization of ventricular myocytes via co-assembly with a broad range of Kv channels (Abbott, 1999; Abbott and Goldstein, 2001; Roepke et al., 2008; McCrossan et al., 2009).

Mechanistically, it is interesting that KChIP2b can alter the effects of T504 phosphorylation, given that the Kv4.3 N-terminal is the established binding site for KChIPs (Bahring et al., 2001; Wang, 2008), but T504 is in the Kv4.3L C-terminal. Importantly, for Kv4.2, a close relative of Kv4.3, both the C- and N-termini have been shown to mediate functional effects of KChIPs (Callsen et al., 2005; Han et al., 2006). The current data, and other recent work from the author (Abbott, 2017), suggest this dual regulation mechanism also applies to Kv4.3L. It would appear that the T504D and T504A mutants do not entirely recapitulate effects of T504 phosphorylation/dephosphorylation, but their effects may offer mechanistic insights. Thus, T504D slowed Kv4.3L-KChIP2b inactivation compared to wild-type or T504A-Kv4.3L-KChIP2b, whereas PMA did not. Therefore, T504D may be able to overcome KChIP2b hindrance to influence Kv4.3L inactivation rate while a phosphorylated threonine at T504 does not. Similarly, mutation of T504 to either A or D negative-shifted the voltage dependence of steady-state inactivation of Kv4.3L-KChIP2b to resemble that of Kv4.3S-KChIP2b. Thus, either mutation hampered the ability of KChIP2b to achieve the more dramatic positive-shift in Kv4.3L voltage dependence facilitated by the 19-residue stretch unique to Kv4.3L. In contrast, PMA did not interfere, again suggesting mutagenesis was more influential than phosphorylation in overcoming effects of KChIP2 in this case. Importantly, while PMA speeded inactivation recovery of homomeric forms of Kv4.3L and Kv4.3S, when KChIP2b was co-expressed, PMA produced substantive speeding only of Kv4.3L-KChIP2b (and not Kv4.3S-KChIP2b). This effect was notable as it reinforces the hypothesis that KChIP2b does not impair T504 phosphorylation. It also raises the question of which phosphorylation site(s) mediated Kv4.3 inactivation recovery speeding in the absence of KChIP2b, which was T504-independent as it occurred for either Kv4.3 splice variant.

### PKC effects on Kv4.3 appear species-dependent

Mixed effects have been found in the past for PKC with Kv4.3. In contrast to the T504-dependent inhibition of human Kv4.3L (and not Kv4.3S, which lacks T504) observed previously, PKC stimulation (10 nM PMA for 30 min) was found to inhibit both long and short forms of rat Kv4.3, and T504A-Kv4.3L, equally (by ~30%). PMA was also previously found to similarly affect rat Kv4.3L and Kv4.3S similarly with respect to normalized conductance and steady-state inactivation (showing no effect) and inactivation kinetics (speeded by ~20%). In contrast, PKC differentially altered the closed-state inactivation (CSI) of either rat isoform, decreasing CSI in Kv4.3S, increasing CSI in Kv4.3L (Xie et al., 2009). Others found that rat Kv4.2, which also lacks the region aligning to the 19-residue stretch in Kv4.3L, and rat Kv4.3 were both inhibited by PKC. The latter was inhibited by 27% by 10 nM PMA after 30 min. However, the Kv4.3 splice variant was not indicated. Rat ventricular *I*_to_ was likewise inhibited (by ~50%) by PKC in that study (Nakamura et al., 1997). In the present study, using human versions of all subunits, the only PKC effect that was Kv4.3 splice variant-nonspecific was the acceleration of recovery from inactivation of homomeric Kv4.3L and Kv4.3S. These differences are difficult to reconcile given that the consensus PKC phosphorylation sites between rat and human Kv4.3 are identical (van der Heyden et al., 2006). Thus, one can only speculate that rat and human Kv4.3L and/or Kv4.3S permit PKC phosphorylation or its effects differently due to other sequence differences outside the consensus sites. For instance, this could involve allosteric effects preventing functional effects, at a distant site, of comparable PKC phosphorylation. Alternatively, there may be Kv4.3 species-specific differences in recruitment of other proteins, endogenous to the expression systems used, that regulate the effects of PKC.

Scholz and colleagues delved further into the molecular basis for PKC regulation of Kv4.3 and native *I*_to_, finding that PKCα is the isoenzyme central to the inhibitory effect (Scholz et al., 2011). In their study, consistent with other reports for rat Kv4.3, they found that the short splice variant, rKv4.3S, was inhibited by PKC (26% after 30 min in 10 nM PMA). In that study, they also examined the effects of PKCα upon Kv4.3S-KChIP2 channels, and found that current was reduced only 10% after 30 min in 10 nM PMA. From this, they concluded that the inhibitory effect of PMA on rKv4.3S was preserved with KChIP2c. While their currents were reduced only to 90 ± 9% of baseline after 30 min, they noted 6% run-up of currents in the absence of PMA over this period. This run-up was not observed in the current study using human subunits; when incubated for 15 min in the biologically inert phorbol ester PDD, hKv4.3S-KChIP2b currents ran down 5%, and Kv4.3L-KChIP2b 12% (Supplementary Figure 1)—more than was observed by Scholz et al. in PMA over 30 min (Scholz et al., 2011). It seems likely that what Scholz et al. observed with PMA and rKv4.3S-KChIP2c was at least a partial inhibition by KChIP2c of the effects of PKC on rKv4.3S. If there were residual functional effects arising from PKC, the difference between this and the present study may again arise from species difference, and the fact that they were observing inhibition even for short Kv4.3 (they did not assess long Kv4.3)—which may well involve a different threonine or serine to the crucial T504 required for PKC inhibition of human Kv4.3L.

### Limitations of the study

*X. laevis* oocytes were used for this study after validation that the primary effect of PKC on human Kv4.3 observed in human ventricular myocytes, i.e., T504-dependent inhibition of peak current (Po et al., 2001), could be recapitulated in oocytes (Figure 1). *Xenopus* oocytes have the advantage of robustness, facilitating long recordings with drugs such as PMA while maintaining cell health and relative lack of non-specific leak. In addition, many matched recordings (positive vs. control) can be completed on a given day using the same fresh preparation of reagent, increasing confidence in the results. *Xenopus* oocytes, because they are individually injected with cRNA, also offer some advantages when performing studies with channels formed from multiple different subunits, as one can better ensure that cRNAs for all the subunits to be studied enter each individual cell. The limitation with *Xenopus* oocytes, as for all heterologous expression systems, is that they lack some of the factors potentially important for reconstituting fundamental native *I*_to_ attributes. In addition, they preclude quantitative assessment of the effects described herein on actual cardiac myocyte action potentials.

In addition, the author did not explore all the different KChIP2 splice variants, or all KCNE genes, expressed in human heart, instead focusing on the most highly expressed (KChIP2b, KCNE2, and KCNE4). Similarly, subunits from other classes that may also modulate Kv4.3 in human heart, including DPPX (Radicke et al., 2005) and Kvβ (Deschenes and Tomaselli, 2002) subunits, were not studied here. Thus, while the studies herein benefit from a reductionist approach to tease out the impact of individual channel subunits on the consequences of Kv4.3 phosphorylation, they may not completely replicate all aspects of native human cardiac *I*_to_. In addition, this work does not resolve the basis of the aforementioned unexplained differences between rat and human Kv4.3 with respect to their regulation by PKC, instead focusing entirely on human subunits.

Finally, there are several different α-adrenergic receptor isoforms in human heart, with α1 being the primary cardioprotective isoform, via PKC activation (Jensen et al., 2014). Further studies, not conducted here, may elucidate whether different PKC isoforms mediate cardioprotective effects vs. inhibition of *I*_to_, and/or whether *I*_to_ inhibition is cardioprotective in preserving aspects of pump function in heart failure, yet predisposes to sudden cardiac death. A precedent for this would be KCNE2, deletion of which predisposes to sudden cardiac death after a severe ischemic insult, yet is protective and reduces infarct size in the context of a less severe ischemic insult (Hu et al., 2014, 2016).

## Author contributions

GA conceived the study, performed the experiments, analyzed the data and wrote the manuscript.

### Conflict of interest statement

The author declares that the research was conducted in the absence of any commercial or financial relationships that could be construed as a potential conflict of interest.

## Abbreviations

*I*_to_: transient outward K^+^ current
KChIP: K^+^ channel interacting protein
Kv channel: voltage-gated potassium channel
MiRP: mink related peptide
PDD: phorbol 4α-Phorbol 12,13-didecanoate
PKC: protein kinase C
PMA: phorbol 12-myristate 13-acetate.
