# Supplementary figures and images for: β Subunits Control the Effects of Human Kv4.3 Potassium Channel Phosphorylation
Source: Front Physiol. 2017 Sep 1;8:646. doi: 10.3389/fphys.2017.00646 (PMC5585193; doi:10.3389/fphys.2017.00646)

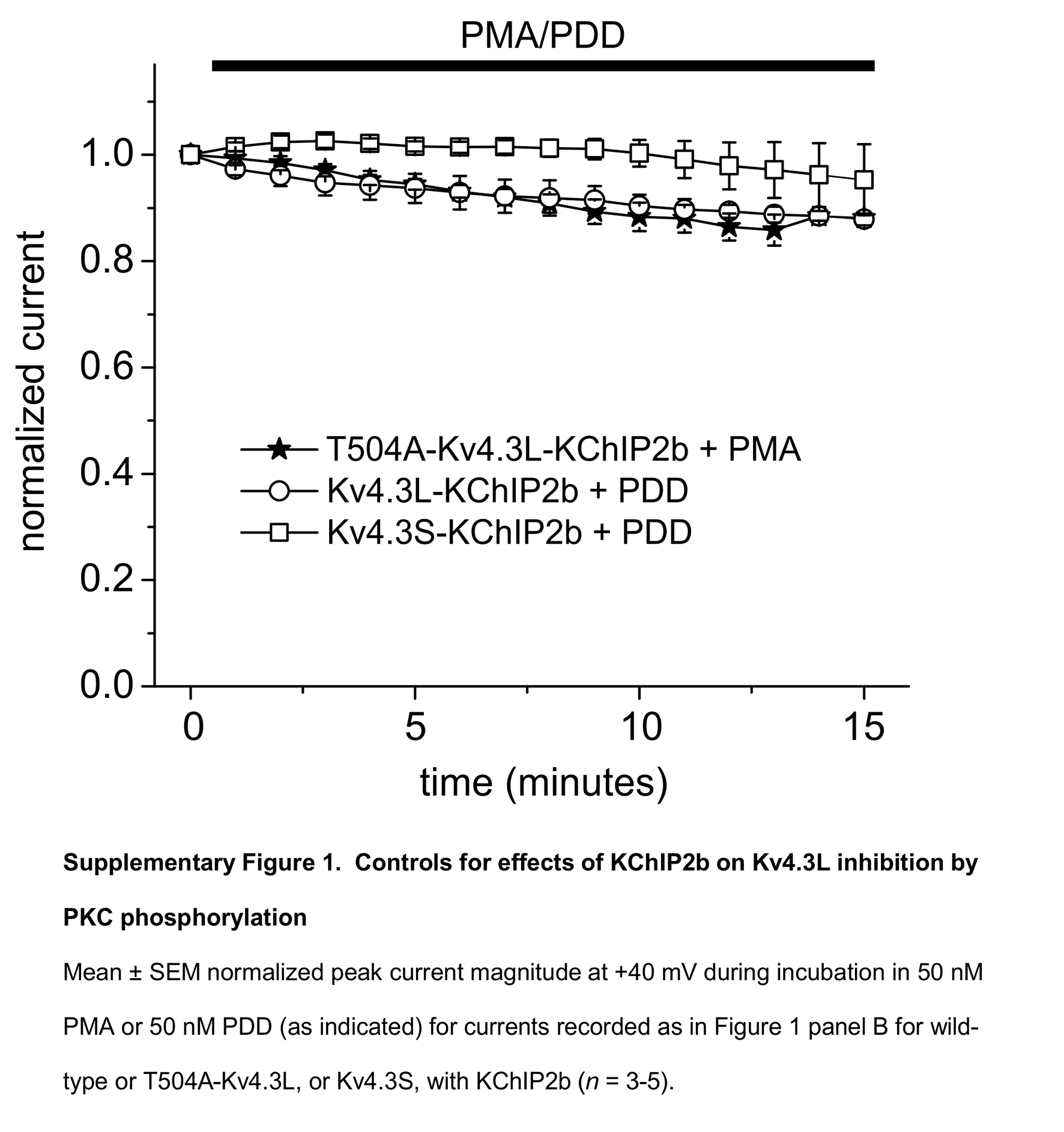

Supplement: Supplementary file 1 [file Image1.tif]

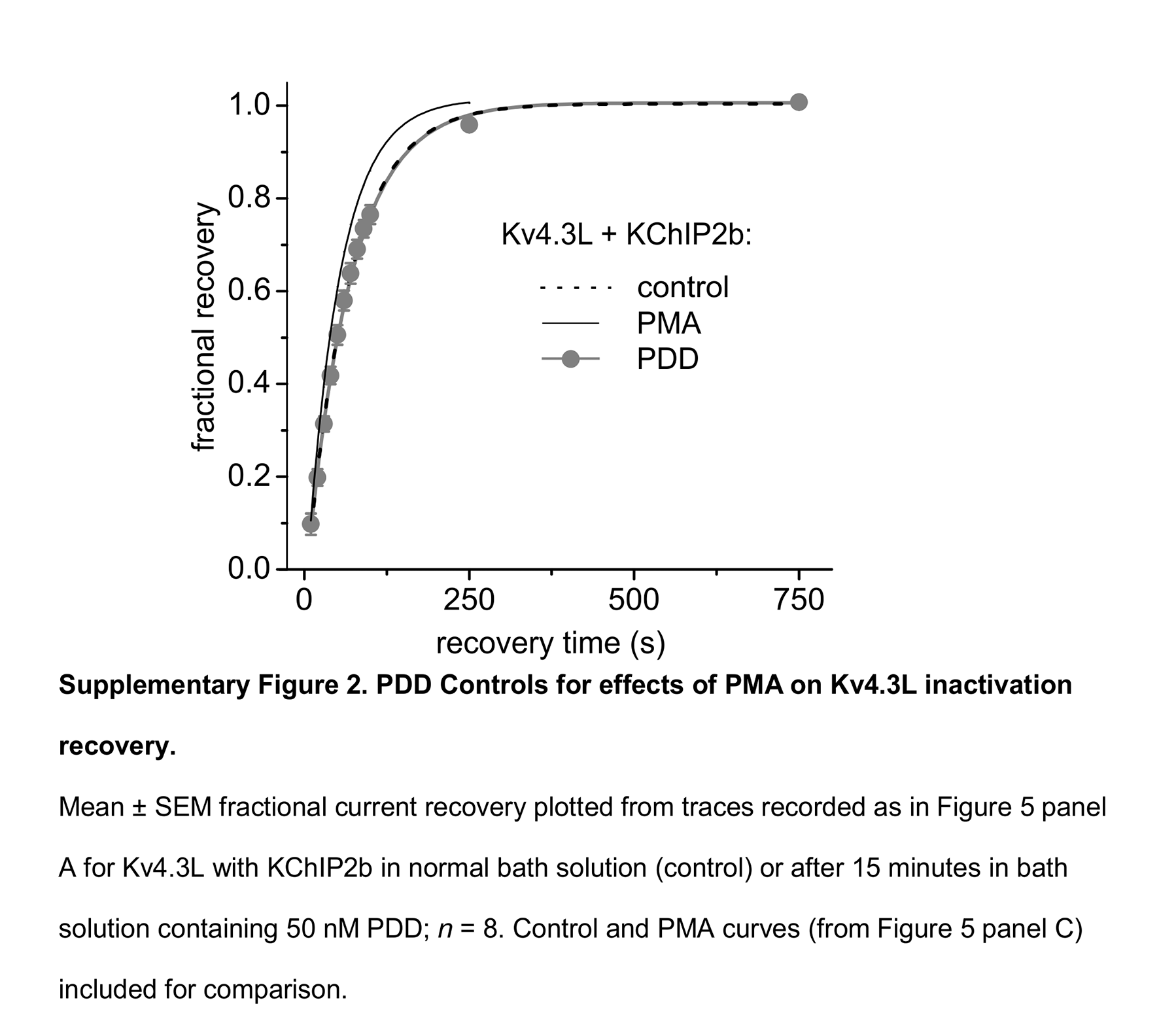

Supplement: Supplementary file 2 [file Image2.tif]

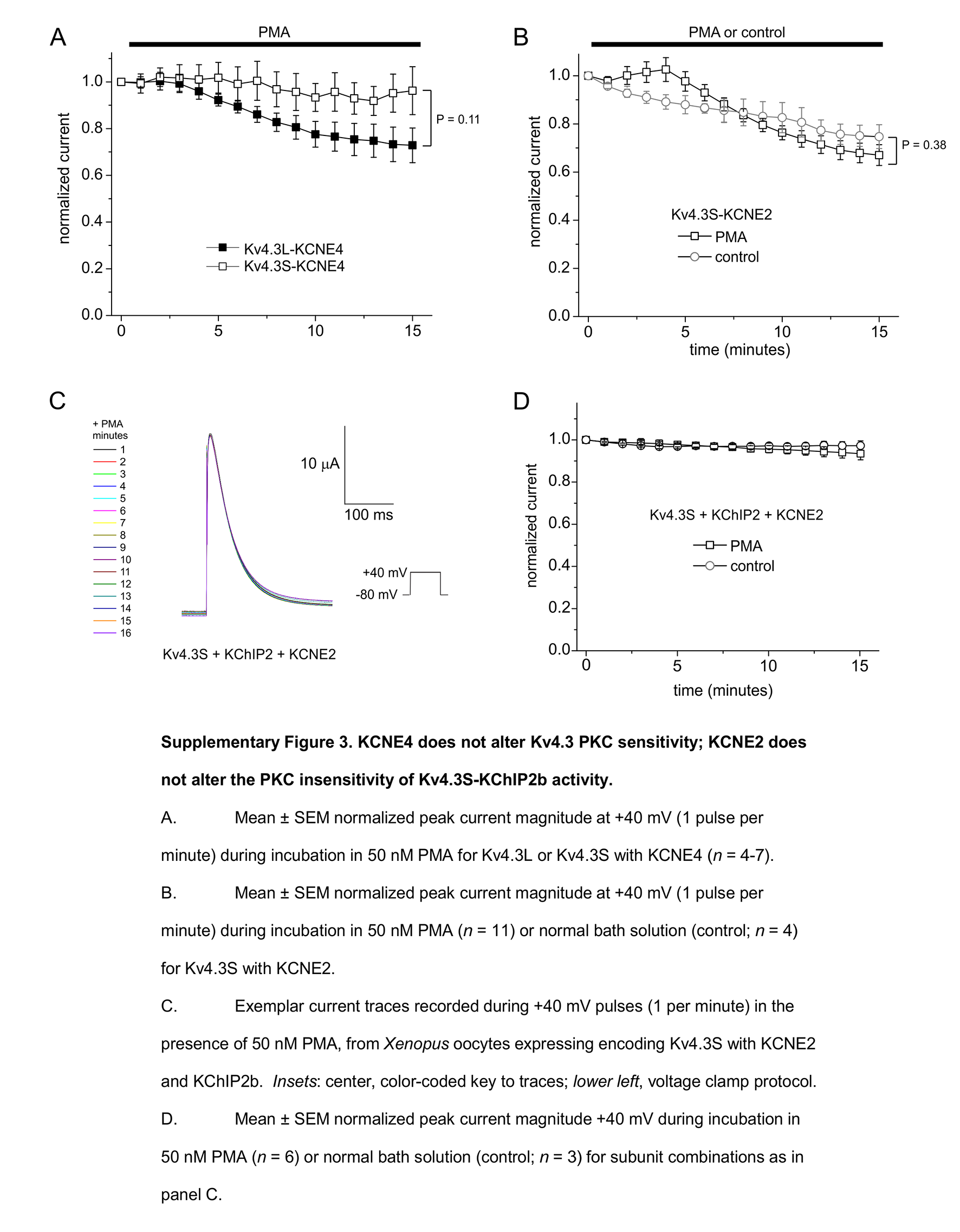

Supplement: Supplementary file 3 [file Image3.tif]
